# Exploratory Study of the Measurement of Geometric Height in 3D Transesophageal Echocardiography as a Predictor of Valve-Sparing Root Replacement for Aortic Regurgitation

**DOI:** 10.3390/jcm13247835

**Published:** 2024-12-22

**Authors:** Shota Yamanaka, Shuichiro Takanashi, Tomoki Shimokawa, Takashi Kunihara

**Affiliations:** 1 Department of Cardiac Surgery, The Jikei University School of Medicine, Tokyo 105-8461, Japan; syamanaka_shi@yahoo.co.jp; 2 Department of Cardiovascular Surgery, Sakakibara Heart Institute, Tokyo 183-0003, Japan; s0715t@gmail.com (S.T.); tshimo@med.teikyo-u.ac.jp (T.S.)

**Keywords:** three-dimensional transesophageal echocardiography, aortic regurgitation, valve-sparing root replacement, composite valve graft replacement, geometric height

## Abstract

**Background**: Valve-sparing root replacement surgery is an alternative strategy for patients with aortic regurgitation with or without aortic root enlargement. A detailed understanding of the mechanisms of regurgitation and the morphology of the aortic root would be beneficial for predicting the feasibility and success of valve-sparing surgery. This is an exploratory study of the measurement of geometric height in 3D transesophageal echocardiography as a predictor of valve-sparing root replacement for aortic regurgitation. **Methods**: Transesophageal echocardiographic findings and long-term outcomes were compared in 124 patients undergoing either valve-sparing root replacement (VSRR group) or composite valve graft replacement (Bentall group) from September 2014 to March 2019. **Results**: The VSRR group was younger and had better left ventricular function than the Bentall group. Three-dimensional transesophageal echocardiography showed that geometric height was significantly larger in the VSRR group. In receiver-operating curve analysis, the cutoff values of geometric height for the feasibility of valve-sparing surgery were 15.9 mm and 19.8 mm in the tricuspid and bicuspid aortic valve, respectively. The overall survival was 98.6% and the freedom from reoperation rate was 89.7% at 5 years in the VSRR group. **Conclusions**: Appropriate patient selection and adequate GH may contribute to the success of VSSR and improve long-term outcomes.

## 1. Introduction

Despite the development of many alternative methods for the management of aortic regurgitation (AR), the gold standard for treatment remains aortic valve replacement (AVR) with mechanical or biological valves. As recent guidelines expanded the indication of surgery for AR to include asymptomatic patients with normal cardiac function but cardiac enlargement [1,2,3], the application of surgical treatment for AR is expected to increase in the future. In AVR, the differences in durability between biological and mechanical valves according to age [4] or valve-related events due to anticoagulation therapy have long been a matter of debate [5,6].

Valve-sparing aortic root replacement (VSRR) is one surgical option for aortic root enlargement with or without AR. Valve-sparing operations for tricuspid and bicuspid valves have yielded excellent results [7,8,9], and it is advantageous to resolve the concerns of anticoagulation and durability associated with mechanical and biological valves, respectively. Nonetheless, as limited durability of biological valves can be solved by transcatheter aortic valve implantation in surgical AVR, VSRR should provide more durability for widespread application. The evaluation of the mechanisms of AR and morphology of the aortic root are crucial to determine the feasibility and success of VSRR. Schäfers et al. proposed tricuspid aortic valve (TAV) and bicuspid aortic valve (BAV) geometric heights (GHs) of 16 mm and 19 mm, respectively, as cutoff values for performing valve-sparing surgery [10]. However, as they were derived from intraoperative data, they were difficult to predict before surgery. The aortic valve leaflet motion is difficult to assess with transthoracic echocardiography (TTE), and contrast-enhanced computed tomography (CT) is associated with concerns about the risks of radiation exposure. Compared to TTE, transesophageal echocardiography (TEE) is more effective in delineating the movement of the aortic valve leaflets, their deterioration, the factors contributing to AR, and the morphology of the aortic root in detail.

To predict the indications for VSRR for AR preoperatively provides a significant advantage in choosing the procedure. The present study was performed to determine the suitability, which is the possibility of controlling AR preoperatively using three-dimensional transesophageal echocardiography (3D-TEE). Furthermore, to verify the appropriateness of our choice of VSRR instead of root replacement using composite valve grafts (Bentall operation), we also examined the long-term outcomes of both surgical procedures.

## 2. Materials and Methods

### 2.1. Patient Selection

A total of 124 patients undergoing elective aortic root replacement surgery between September 2014 and March 2019 were enrolled in this study. Patients with infective endocarditis, acute aortic dissection, a history of previous cardiac surgery, unicuspid or quadricuspid aortic valve, AR due to rheumatic changes, relevant aortic stenosis, age over 70 years, or who did not undergo preoperative TEE were excluded. Patients who underwent concomitant cardiac surgery were not excluded. We separated patients into two groups who underwent VSRR or Bentall operation and compared patient characteristics, TTE parameters, and 3D-TEE parameters.

### 2.2. Surgical Methods

The application of VSSR or Bentall operation was decided by the surgeon intraoperatively. All operations were performed with full median sternotomy. Cardiopulmonary bypass was established by ascending aortic and bicaval cannulation. The aorta was cross-clamped and cardiac asystole was achieved using intermittent selective and retrograde cardioplegia. If circulatory arrest was necessary for ascending aorta replacement, the core temperature, including bladder or rectal temperature was cooled to 25 °C, and retrograde cerebral perfusion was used with reference to central venous pressure. VSRR was performed by an aortic root remodeling technique with an external flexible ring to perform annuloplasty as described previously by Lansac et al. [11]. Briefly, all three sinuses of Valsalva were replaced using either a neo-Valsalva graft (Gelweave Valsalva; Terumo, Tokyo, Japan) or a tube graft (J Graft; Japan Lifeline, Tokyo, Japan) with three tongue-shaped incisions matching their shape. The diameter of the AVJ was used as a reference for determining the size of the artificial graft intraoperatively. When the aorto-ventricular junction (AVJ) diameter was larger than 28 mm or the GH was greater than 20 mm in the TAV, a 28 mm prosthesis was used, and if the AVJ diameter was smaller than 28 mm or the GH was shorter than 20 mm, a 26 mm prosthesis was used. External flexible ring annuloplasty was performed using the same graft cut into 5 mm pieces, which were fixed at the basal ring level with 6 pledgeted mattress sutures (Tefdesser; Crownjun, Chiba, Japan). Effective height (eH) was measured in all patients in the VSRR. The eH is the vertical distance from the bottom of the aortic valve ring to the tip of the valve leaflet, measured with a dedicated sizer. After prosthetic replacement of the aortic root, all valve cusps were adjusted to have an eH greater than 8 mm in the TAV and in the non-fused cusp in the BAV. Bentall operation was performed using a handmade composite valve graft with either mechanical or biological prosthesis. The Bentall procedure was performed with pledgeted mattress sutures similar to VSRR, and the composite graft was sutured in the intra-annular position using an everting mattress technique with a 10 mm felt. Coronary arteries were reconstructed with the Carrel patch technique in all cases.

### 2.3. Echocardiographic Parameters and Measurement

**Figure 1:**
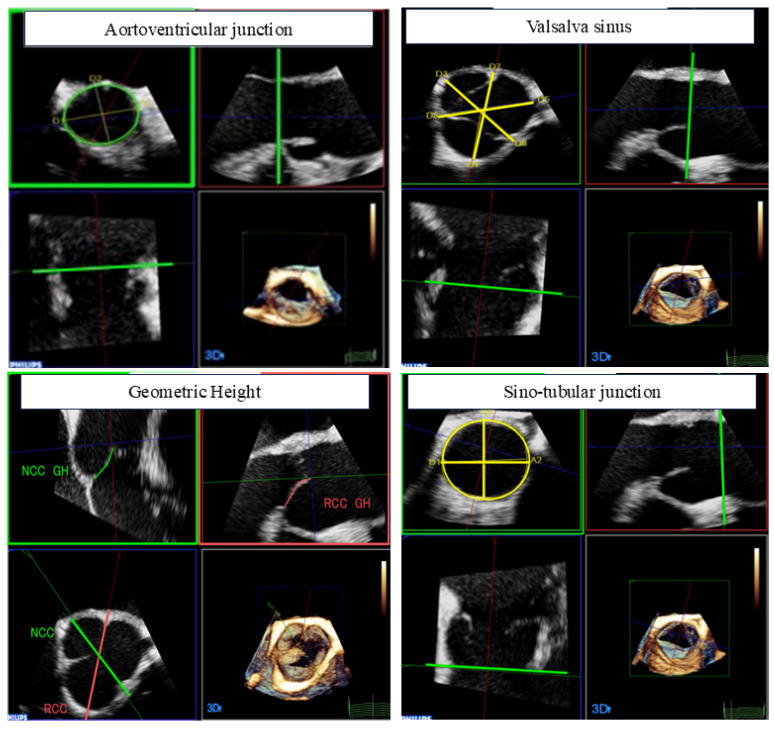
The measurement of 3D-TEE parameters. Aortoventricular junction, sinus of Valsalva, and sino-tubular junction were measured in the systolic phase, and the geometric height was measured in the diastolic phase. GH; geometric height, NCC; non coronary cusp, RCC; right coronary cusp.

Transesophageal echocardiography was performed using an EPIQ system with an X7-2t transducer (Philips Medical Systems, Andover, MA, USA). Echocardiographic images were transferred to and stored in a computer to create 3D images for off-line analysis using Xcelera (Philips Medical Systems). Comparisons were performed based on preoperative 3D-TEE parameters, (AVJ), GH, diameters of the sinus of Valsalva, and sino-tubular junction (STJ) were measured with multiplanar reconstruction (MPR). Several 3D-TEE parameters were measured as shown in Figure 1. The AVJ was defined as a virtual ring with anatomical anchor points at the nadir of each of the attachments of the aortic leaflets in the systolic phase. The GH was the cross-section cutting of each cusp orthogonally; it was extracted and measured from the hinge point to the cusp tip in diastolic phase. The sinus of Valsalva and STJ was measured in the short-axis view by extracting the appropriate cross-section from a long-axis view in the systolic phase. The GH was divided into tricuspid and bicuspid valves and compared between the two groups.

### 2.4. Follow-up and Ethical Statement

Long-term outcomes were evaluated with an average follow-up of 43 ± 20 months. The follow-up duration was significantly different between the two groups: VSRR group, 46 ± 19 months; Bentall group, 33 ± 20 months (*p* < 0.01). The institutional review boards of both participating institutions approved this study (approval numbers: 32-076 (10151) and 19-086). The requirement for written informed consent was waived due to the retrospective nature of the study. The manuscript was written according to the strengthening of the reporting of the observational studies in epidemiology (STROBE) statement.

### 2.5. Statistical Analysis

Statistical analyses were performed using SPSS ver. 28 (SPSS Inc., Chicago, IL, USA). Normally distributed continuous variables are expressed as the mean ± standard deviation, and nonnormally distributed continuous variables are expressed as the median with interquartile range. Student’s *t* test or Wilcoxon’s signed rank test were used for the comparison of continuous variables between the two groups, as appropriate. Categorical variables were expressed as number and percentage and were compared between the two groups using the Chi-square test. Survival rate and reintervention-free rate were calculated using the Kaplan–Meier method and compared with the log-rank test. In all analyses, *p* < 0.05 was taken to indicate statistical significance.

## 3. Results

### 3.1. Preoperative Data

The preoperative characteristics are summarized in Table 1. The VSRR group was significantly younger (50 ± 13 years vs. 59 ± 10 years, respectively, *p* < 0.01) and had significantly better left ventricular ejection fraction (55.4% ± 8.0% vs. 50.9% ± 9.3%, respectively, *p* = 0.01) than the Bentall group. Compared to the VSRR group, the Bentall group included more women (10.3% vs. 22.2%, respectively, *p* = 0.11) and more patients with atrial fibrillation (2.1% vs. 11.1%, respectively, *p* = 0.07), but the differences were not significant. The BAV was observed in approximately one-third of patients in both groups (34.0% and 29.7%, respectively, *p* = 0.67).

In 3D-TEE measurements, there were no significant differences in AVJ area, the diameter of the sinus of Valsalva, or the STJ diameter between the groups (Table 2). Only the GH was significantly longer in the tricuspid aortic valve of the VSRR group compared to the Bentall group (Table 3 and Table 4).

### 3.2. Operative Data

Operative data are shown in Table 5. Cardiopulmonary bypass time (226 ± 46 min vs. 191 ± 63 min, respectively, *p* < 0.01) and aortic cross-clamp time (184 ± 38 min vs. 151 ± 52 min, respectively, *p* < 0.01) were significantly longer in the VSRR group than the Bentall group. For VSRR, central plication was performed in the majority of patients. The autologous pericardium was used in one-third of the patients when only one leaflet was degenerated.

**Figure 2:**
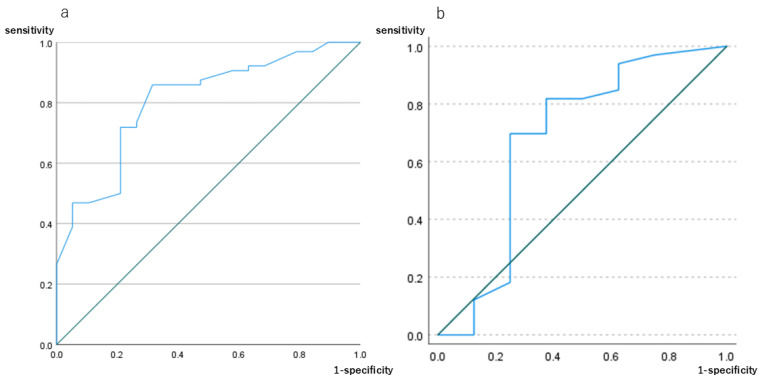
ROC analysis of geometric height. (**a**) Tricuspid valve, cutoff 15.9 mm (shortest GH), area under the curve (AUC): 0.81, *p* < 0.01, sensitivity and specificity were 0.859 and 0.684, respectively. (**b**) Bicuspid valve cutoff 19.8 mm (non-fused cusp), AUC: 0.67, *p* < 0.01, sensitivity and specificity were 0.818 and 0.625, respectively.

In receiver-operating curve (ROC) analysis, the optimal cutoff values of GH for detecting the suitability of VSRR were 15.9 mm in the TAV and 19.8 mm in the BAV (Figure 2).

### 3.3. Postoperative Data

**Figure 3:**
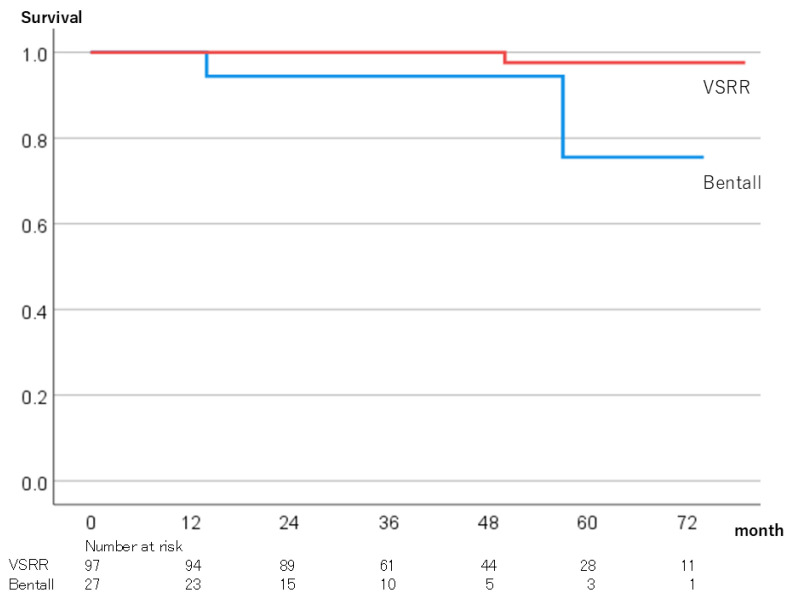
Overall survival rates. VSRR; Valve-sparing aortic root replacement.

**Figure 4:**
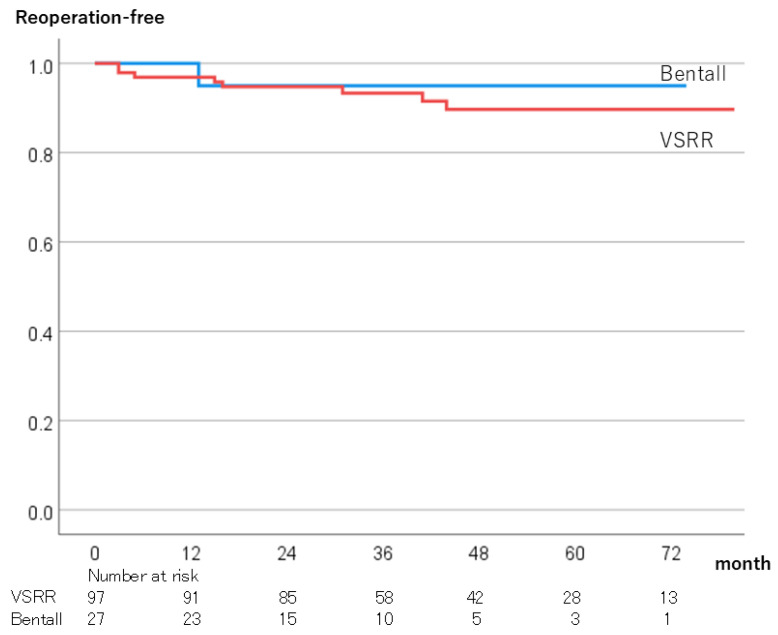
Reoperation-free rates. VSRR; Valve-sparing aortic root replacement.

Overall survival rates at 5 years were 97.6% in the VSRR group and 75.6% in the Bentall group (*p* < 0.01) (Figure 3). The reoperation-free rates at 5 years were 89.7% in the VSSR group and 95.0% in the Bentall group, and the difference was not significant (*p* = 0.63) (Figure 4).

## 4. Discussion

AVR with biological or mechanical valves has been the gold standard surgical treatment for AR. In groups matched for age and sex, the overall survival after AVR was reported to be the same as in the general population [12]. In a meta-analysis comparing aortic valve plasty (AVP) and AVR, in-hospital mortality and long-term event rates were lower in AVP than AVR [13]. Although use of a biological prosthesis requires no anticoagulation therapy, it is associated with a risk of structural valve deterioration or prosthesis/patient mismatch. Younger patients with biological valves have a lower survival rate than the general population, despite the low risk of reoperation [14]. In addition, the use of a mechanical valve is a risk factor for bleeding and thromboembolic events due to anticoagulation therapy, with a significant impact on survival [15].

VSRR is one of the surgical options for aortic root enlargement with or without AR. Recent studies have demonstrated good late results of VSRR for patients with annuloaortic ectasia, BAV, and isolated AR [8,9,16].

The valve-in-valve procedure (transcatheter AVR in surgical AVR) has been performed in recent years, but the late outcomes are unclear. The durability of biological prostheses has improved, so that valve-sparing surgery should also provide longer durability and become more widely adopted. For this purpose, the accurate preoperative selection of optimal candidates for valve-sparing surgery is of paramount importance, which is the primary reason for initiating this study. In addition, to verify the validity of our decision to select valve-sparing surgery, we also examined the long-term outcomes of both surgical procedures.

Schäfers et al. proposed that cusp height is one of the most important parameters for performing valve-sparing surgery. They stated that the method of measurement of GH is important and that it was underestimated in previous methods [10].

In this study, the VSSR group was significantly younger and had better left ventricular function but required longer procedure times than the Bentall group. These differences may have been due to surgeon-dependent decisions on the procedure with the assignment of high-risk patients, such as those of advanced age or with low cardiac function, to the Bentall group. In other studies, procedure time was also significantly longer in the VSRR group, which was a natural result considering the complexity of the procedure [17,18].

We used 3D-TEE in this study. TEE allows a more detailed evaluation of leaflet motion and root structure than TTE, and 3D-TEE allows the accurate evaluation of GH. This article also states that this is particularly useful for patients who are difficult to diagnose with imaging, such as those with obesity or chronic lung disease [19]. In this study, there were no differences in the diameter of AVJ, the sinus of Valsalva, and STJ, so there were also no differences in the surgical indications for aortic root replacement between the two groups. Only the GH was significantly longer in the TAV in the VSRR group, but there was no difference in the BAV. This may have been due to the small number of cases with a BAV. When the leaflet motion and GH are adequate, root replacement with a prosthesis of appropriate size is important for the success of VSRR. In ROC analysis, the optimal GH cutoff values for determining suitability for VSRR were 15.9 mm in the TAV and 19.8 mm in the BAV, which were close to the values proposed by Schäfers et al. [10].

There was a significant difference in the long-term survival rate between the two groups, which was likely due to the age difference between the two groups. The 5-year survival rate of the VSRR group was 97.6% ± 2.6%, which was similar to previous studies [17,18]. There was no difference in the reoperation-free rate between the two groups. The 5-year reoperation-free rate of the VSRR group was 89.7% ± 3.6%, which was slightly lower than in previous studies [17,18]. This may have been due to the use of autologous pericardium in 35.1% of the cases in this study. In our study, differences in preoperative background were associated with differences in long-term survival. In other studies that included propensity score matching, the long-term survival and reintervention rates were better for VSRR than Bentall operation with biological valves [20].

The 3D-TEE measurements showed significant differences only for GH in both groups, and ROC analysis showed a cutoff value of GH that predicted VSRR. Appropriate patient selection and adequate GH may contribute to the success of VSSR and improve long-term outcomes.

## 5. Limitations

This study had several limitations. The sample size was small, and this was a retrospective and nonrandomized study, so considerable inhomogeneity between the two groups remained a matter of concern. It would be ideal to perform matching between the groups, but this would have further reduced the sample size. This study is subject to selection bias, unmeasured and residual confounding, informative censoring, and possible Type I and Type II errors. The choice of surgery was left to the surgeon’s decision, and there were no selection criteria for determining which procedure to perform. Non-anatomical factors such as patient age, general condition, and comorbidities may have influenced the choice of procedure. The follow-up period was also relatively short, so long-term outcomes should be analyzed in future studies. Nonetheless, the detailed evaluation of the aortic root geometry in this study will contribute to the further success and widespread adoption of valve-preserving surgery.

## 6. Conclusions

The preoperative measurement of GH for VSRR for AR is important to determine the procedure. It may be useful to measure the geometry of the aortic root using an appropriate method to ensure acceptable long-term durability after valve-preserving surgery.

## Figures and Tables

**Table 1:** Preoperative characteristics.

Variables	VSRR (n = 97)	Bentall (n = 27)	*p* Value
Age (years)	50 ± 13	59 ± 10	<0.01
Men, n (%)	87 (89.7%)	21 (77.8%)	0.11
BSA (kg/m^2^)	1.79 ± 0.16	1.80 ± 0.22	0.84
Atrial Fibrillation, n (%)	2 (2.1%)	3 (11.1%)	0.07
Bicuspid Valve, n (%)	33 (34.0%)	8 (29.7%)	0.67
NYHA, n (%)			0.11
I	69 (71.1%)	16 (59.3%)	
II	21 (21.6%)	7 (25.9%)	
III	6 (6.2%)	4 (14.8%)	
TTE Parameter			
EF (%)	55.4 ± 8.0	50.9 ± 9.3	0.01
LVDd (mm)	61.0 ± 8.7	62.5 ± 8.2	0.43
LVDs (mm)	43.7 ± 8.9	46.3 ± 8.6	0.19

BSA, body surface area; TTE, transthoracic echocardiography; EF, ejection fraction; LVDd, left ventricular end-diastolic dimension; LVDs, left ventricular end-systolic dimension.

**Table 2:** Preoperative 3D-TEE of aortic root.

Variable	VSRR (n = 97)	Bentall (n = 27)	*p* Value
Area of AVJ (mm^2^)	625 ± 164	558 ± 139	0.06
Sinus of Valsalva (mm)	45.2 ± 7.9	43.0 ± 5.5	0.10
STJ (mm)	39.7 ± 9.5	38.2 ± 6.8	0.36

AVJ, aorto-ventricular junction; STJ, sino-tubular junction.

**Table 3:** Preoperative 3D-TEE of GH in tricuspid aortic valve.

	VSRR (n = 64)	Bentall (n = 19)	*p* Value
GH of RCC (mm)	18.1 ± 2.3	16.3 ± 2.5	<0.01
GH of LCC (mm)	18.4 ± 1.9	15.6 ± 2.0	<0.01
GH of NCC (mm)	19.3 ± 2.2	16.5 ± 1.7	<0.01
GH of the shortest (mm)	17.3 ± 1.9	15.0 ± 2.0	<0.01

GH, geometric height; RCC, right coronary cusp; LCC, left coronary cusp; NCC, non-coronary cusp.

**Table 4:** Preoperative 3D-TEE of GH in bicuspid aortic valve.

	VSRR (n = 33)	Bentall (n = 8)	*p* Value
GH of non-fused cusp(mm)	21.1 ± 2.0	19.6 ± 3.3	0.09

GH, geometric height.

**Table 5:** Operative data.

Variable	VSRR (n = 97)	Bentall (n = 27)	*p* Value
Operative time (min)	339 ± 36	308 ± 88	0.06
CPB time (min)	226 ± 46	191 ± 63	<0.01
ACC time (min)	184 ± 38	151 ± 52	<0.01
Central plication, n (%)	71 (73.2%)		
Autologous pericardium, n (%)	34 (35.1%)		

CPB, cardiopulmonary bypass; ACC, aorta cross clamp.

## Data Availability

The data that support the findings will be made available on request by the corresponding author.
